# Reply to “A Synopsis of a Case of Hemorrhoids”

**Published:** 1890-05

**Authors:** E. W. Berridge

**Affiliations:** London, England


### REPLY TO "A SYNOPSIS OF A CASE OF HEMORRHOIDS."

E. W. Berridge, M. D., London, England.

I do not usually take any notice of anonymous attacks, especially when they only show the ignorance or malice of their author, but as my silence might be misconstrued to the detriment of true Homœopathy, I will briefly reply.

(1.) My critic accuses me of prescribing *Phosph.* on October 16th, on the *one* symptom that the patient was worse when sitting or lying. He admits that Phosphorus is the only medicine recorded under that symptom in the *Repertory*, but yet refuses to look upon it as a keynote, and evidently thinks I ought not to have prescribed it. This is the first proof how carelessly he has read my paper. If he will again refer to the report on page 40 he will find that the patient wrote to me, and that the Phosphorus "much relieved her, entirely removing the following symptoms." To ordinary minds this would convey the idea that there were other symptoms indicative of the remedy besides the very peculiar one which I quoted *verbatim.* This was the case, though as I only subsequently copied into my case-book the very peculiar symptom recorded, I cannot now inform him what they were; he will doubtless find them in the *Materia Medica.*

(2.) He declares that in painful piles "it is almost impossible to sit down without pain, and lying down needs just as much care." I will only say that in my interleaved copy of Lippe's *Repertory* I have added the following symptoms (whether pathogenetic or clinical I cannot now say):

Hemorrhoids better when lying—*Am-c.*, *Arsen.*
" better by hard pressure—*Kali-c.*
" better by riding—*Kali-c.*
" better by sitting—*Arsen.*, *Laches.*

These *facts* hardly coincide with his *theories.*

(3.) He says *Phosph.* "does not give shooting pains in the vagina near orifice." Possibly not, as far as our provings show at present, and, therefore, all the more reason for reporting the complete cure of the very peculiar symptom which has caused him so much concern. But *Phosphorus* has (*Lippe's Text-book*, p. 525), "stitches upward in the vagina into the pelvis."

(4.) He says: "As the wall of the vagina posteriorly is the wall of the rectum anteriorly it seems (!) to me that the diagnosis would have been more scientific and correct if the vagina had not been mentioned, as it had probably (!) little or nothing to do with the case." I would remind my critic that the pains in question were expressly stated to be "in vagina near orifice," and that between the orifice of the vagina and rectum is usually situated an anatomical region called the perineum. Besides, I may as well inform my critic that my patient was a very intelligent lady, a graduate in arts of a university, and quite sufficiently acquainted with anatomy to know the difference between her rectum and her vagina. But what does my critic make of the concluding part of the symptoms: "Needle pains in rectum, also near orifice, sometimes alternating with the vaginal pains, but more often independent"? Does not this graphic and minute description show that my patient could not have made the blunder of which she is accused?

(5.) He says piles "may pass off as this did for a time, by an increased action of the liver, producing bilious diarrhœa." But as there was no bilious diarrhœa in this case, but a contrary constipation, it is difficult to understand his argument.

(6.) He says the symptoms "all again point strongly to *Sulphur*, and yet this poor lady is kept suffering more or less from the 16th of October to 27th of December, when we are told her *climax* of pain came, and her *physician* saw he had committed *a fatal error* (why all these italics?) in giving *Kali-carb.* and then makes another by giving Hamamelis$^{cm}$." Considering that I called this case, treated more than six years ago, "a confession and a warning," it is rather rough on me to accuse me of the errors I had admitted; but my critic has again showed that he has read the case very carelessly. As to whether *Sulphur* was the *simillimum* I need not enter; the *Materia Medica* is open to the perusal of all the readers of THE HOMŒOPATHIC PHYSICIAN. But he has overlooked the fact that the patient was not entirely under my care; therefore, I am only responsible for my own prescriptions. Moreover, seeing that the patient was greatly relieved of the piles by *Phosph.* prescribed on October 16th, the accidental attack of sciatica removed by *Ammon.-carb.*, prescribed on October 28th, improvement commencing two or three days after the first dose of *Kali*, the patient's own words on December 19th being "feel myself improving from hour to hour;" to charge me with having "kept this poor lady suffering more or less from 16th October to 27th December" is scarcely an accurate way of putting it. Furthermore, the italicized word "*climax*" is scarcely the appropriate phrase in which to describe the aggravation following a marked improvement as the result of over-medication. Also, I did not confess a "*fatal error*" in giving *Kali-carb.*, which I still believe to have been the *simillimum*, but in repeating the dose after such a marked improvement. Neither did I confess to "*a fatal error*" in giving *Hamamelis;* I merely suggested that I was not now sure if it would not have been better simply to allow the over-action to pass off without interference. My critic has evidently forgotten the advice of an American celebrity, "First be sure you are right, then go ahead."

(7.) He says, "more than three months' treatment and absolutely nothing done." This is simply an insult to the understanding of his readers, if he thinks they can be so deceived by his reckless and unauthorized statements. Part of this time the patient was not under my care, and, moreover, had been subjected to treatments I disapproved of, as I clearly stated in my report; in spite of these drawbacks, however, very much good was done; and if I had not fallen into the error of giving too much medicine I believe the *Kali* would have completed the cure (I may here mention, as I omitted it in my report, that the patient would not wean her baby as soon as I advised).

(8.) To his remark on the symptom of *Silicea*, "stool feels as if it slipped back," I need answer little. This symptom has been so thoroughly established as a keynote for this medicine (whatever the "probability" of its cause may be) that it would be waste of time to quote authorities. But my critic seems to doubt whether *Silicea* did anything, or whether the relief was simply the effort of nature, I am not concerned about the matter; it is quite possible that the bowels would have got into proper order when allowed to act naturally, but seeing that a keynote of *Silicea* was present, and that the patient had fear of suicide I consider I did right to give it. But the most amusing part of his criticism is that he states that it took me "*three months to find the remedy*" for symptoms which had only existed for eleven days before I prescribed, and for which I prescribed the very first day they were reported to me. This logic is quite beyond me; perhaps the "Christian Scientists" can explain.

(9.) Finally he gives his theory of the case. He suggests the baby was "*a thirteen months' child*" (I am altogether too obtuse to see where this joke comes in), that "the piles gradually went away of themselves" ("gradually" is very inaccurate); and "the treatment they received not affecting them in the least" (which is simply at utter variance with the facts of the case). "So much for that *physician* who seems to think he can teach better and wiser men than himself." If my critic here refers to himself, he may rest assured I shall never think of ever accomplishing that feat till he has trained himself in the habits of careful reading and accurate quotations.

I have endeavored to find the true *animus* of his criticism, and I think I have found it in his complaint that it almost seems that he would rather have let the poor lady suffer than give anything short of a CM potency. "And this in the face of the fact that I successfully gave her *Amm.-carb.*$^{2300}$." But we all know the proverbial effect of a red rag on the male of the bovine species, and as he calls himself "Buffalo," he has evidently been so infuriated by this CM potency that he has closed his eyes, lowered his head, and charged down furiously upon me. However I think I have escaped being tossed on his horns, while he is left impaled on the horns of a dilemma, the charge of either careless criticism or intentional malice.

Let me hope that if he pursue this controversy further, he will drop his disguise. Whoever he may be, I know that he is not one of the I. H. A., and for the opinion of the anti-Hahnemannians I have no regard whatever.

